# Acyclic graphs to define models of relationships between human cytomegalovirus, cardiovascular disease and all-cause mortality in UK Biobank

**DOI:** 10.1098/rstb.2024.0415

**Published:** 2025-11-06

**Authors:** Robert Doorly, Amanda Y. Chong, Elizabeth Hamilton, Thomas J. Littlejohns, Julian C. Knight, Seilesh Kadambari, Paul Klenerman, Alexander J. Mentzer

**Affiliations:** ^1^ School of Clinical Medicine, University of Cambridge, Cambridge CB2 0SP, UK; ^2^ Centre for Human Genetics, University of Oxford, Oxford OX3 7BN, UK; ^3^ University of Oxford Nuffield Department of Population Health, Oxford OX3 7LF, UK; ^4^ Chinese Academy of Medical Science (CAMS) Oxford Institute, Oxford OX3 7BN, UK; ^5^ Department of Paediatric Infectious Diseases and Immunology, Great Ormond Street Hospital for Children, London WC1N 3BH, UK; ^6^ Peter Medawar Building for Pathogen Research, University of Oxford, Oxford OX1 3SY, UK; ^7^ Translational Gastroenterology Unit, Nuffield Department of Clinical Medicine, University of Oxford, Oxford OX3 7BN, UK; ^8^ NIHR Oxford Biomedical Research Centre, Oxford University Hospitals NHS Foundation Trust, Oxford OX3 9DU, UK

**Keywords:** cardiovascular disease, stroke, myocardial infarction, prospective study, UK, cytomegalovirus

## Abstract

Human cytomegalovirus seropositivity has shown varying levels of association with cardiovascular disease and all-cause mortality in previous studies. This study uses updated data from the UK Biobank to test these associations. The association between human cytomegalovirus seropositivity and outcomes of incident cardiovascular disease, ischaemic heart disease, stroke and all-cause mortality was determined using multivariate Cox proportional-hazards models in 8740 UK Biobank participants aged 40–69 years. Two directed acyclic graphs depicting the hypothesized relationship between human cytomegalovirus infection (either in childhood or adulthood) and cardiovascular disease/all-cause mortality controlled the selection of biological and socioeconomic confounders to be adjusted for in each model. Human cytomegalovirus seropositivity was not significantly associated with cardiovascular disease, ischaemic heart disease, stroke or all-cause mortality when applying either the fully adjusted adulthood or childhood infection models. We found no significant association between human cytomegalovirus seropositivity and any of the measured outcomes. Further research is expected to include larger sample sizes, younger participants and more ethnically diverse cohorts.

This article is part of the discussion meeting issue ‘The indirect effects of cytomegalovirus infection: mechanisms and consequences’.

## 1. Introduction

Human cytomegalovirus (HCMV) is a ubiquitous and highly transmissible herpes virus with seroprevalence estimates varying from 60 to 85% in the UK [1] to ≥90% in adults in less developed countries [2]. Exposure to HCMV may occur *in utero*, perinatally, or later in life through infected bodily fluids (usually saliva or urine) or blood transfusion [1,3]. Primary HCMV infection is typically asymptomatic in immunocompetent individuals owing to the robust immune response elicited [4]; however, in immunocompromised individuals, HCMV is associated with severe end-organ disease [5], and congenital infections can lead to hearing loss and blindness [6]. HCMV establishes a lifelong presence that appears to have some elements of both chronic productive infection and latent infection with regular subclinical reactivation [7]. It has been hypothesized that HCMV infection may be associated with elevated risk of chronic diseases including cardiovascular disease (CVD) [8], cancer [9–11] and all-cause mortality. Detection of HCMV antigen and DNA in atherosclerotic vessels of the human cardiovascular system [12–14] suggests that infection could drive atherogenesis via inducing chronic inflammation or dysregulating vascular function [15]. If HCMV were proven to be a driver of CVD or other chronic diseases, actively reducing its transmission or downstream inflammatory reactivation through vaccination, could have a positive effect at a wider population health level.

Studies investigating the relationship between prior HCMV infection, defined as cross-sectional antibody (IgG) seropositivity, and all-cause mortality show mixed results. Three large cohort studies [7,16,17] all showed a significant increase in all-cause mortality among populations in the USA [7,16] and UK [17], even when adjusting for age, sex and multiple other potential confounders. The UK-based EPIC-Norfolk study [17] also demonstrated an association between higher quantitative IgG antibody levels against HCMV and increased all-cause mortality. However, two further studies in the Netherlands and Denmark combined, and in Belgium, respectively, did not show any association between HCMV infection and all-cause mortality among older white populations [18,19].

The increases in all-cause mortality for HCMV-positive patients in the USA NHANES-III (Third National Health and Nurition Examination Survey) [7] and UK EPIC-Norfolk studies [17] were both driven by a rise in CVD-related deaths. A meta-analysis [8] of 10 prospective studies concluded that HCMV seropositivity slightly increased the risk of developing CVD; however, several of the included studies lacked sufficient control for confounders, had short follow-up times or the populations studied had significant comorbidities at baseline. A recent prospective study [20] investigated the relationship between HCMV and incident CVD among 8500 middle-to-early older-aged participants in the UK Biobank (UKB), finding no association with either ischaemic heart disease (IHD) or stroke when adjusting for confounders encompassing socioeconomic background, general physical health and activity. Notably, findings were also null in the minimally adjusted models including age and sex alone. The decision to include any of these covariates, including even age, can remain contentious. At the simplest level, if individuals acquired HCMV in earlier childhood, there is the theoretical possibility that the duration of the inflammatory response may be longer than for those individuals who acquired infection in adulthood, increasing the risk of downstream inflammatory sequelae. Since the UKB HCMV data are based on a cross-sectional sample in time, the age at infection is unknown. However, the depth and breadth of data available from UKB offer an opportunity to explore different models assuming different ages of exposure that can be defined using careful selection of covariates through direct acyclic graphs (DAGs). DAGs demonstrate interactions within biological systems, comprising variables (exposure, outcome of interest and biological and socioeconomic factors) and arrows that depict known or researcher-hypothesized causal relationships between the variables. Analysing relationships between the variables in DAGs can assist researchers in adjusting their analysis to limit bias. Confounding variables are associated with both exposure and outcome of interest, so should be adjusted for, while mediators lie on the path between exposure and outcome, so should not be controlled for. Furthermore, inclusion of variables, such as smoking status, can be questioned, even if they are both associated with HCMV and CVD risk. Even if smoking is associated with HCMV risk, there is a biological plausibility that smoking may be a collider, associated causally with CVD risk but only associated through other correlated risks with HCMV risk.

Here, we present an updated analysis of UKB data on HCMV and CVD and all-cause mortality endpoints, using a larger number of accrued events. Furthermore, we have explicitly modelled different variables as either confounders or mediators using DAGs, attempting to determine whether HCMV exposure in childhood or adulthood may contribute to later disease or mortality outcomes and examining the differential effect of including or stratifying by age and smoking status in testing for HCMV chronic disease risk.

## 2. Methods

### (a) Population

Participants were selected from the UKB, a prospective population-based cohort study following middle-aged to older adults recruited between 2006 and 2010. Approximately, 9.2 million adults aged 40−69 years at the time and living within 25 miles of one of the 22 baseline assessment centres were mailed invitations to join the study, recruiting just over 500 000 participants. Detailed lifestyle, environment and medical history for each patient were assessed through questionnaires and verbal interviews. Participants also underwent physical tests and had samples taken of blood, urine and saliva. One lakh participants were then invited to re-attend centres from 2012 to 2013 to be assessed again, of which 20 000 attended. Ethical approval for the study was provided by the National Information Governance Board for Health and Social Care and the National Health Service North West Multicentre Research Ethics Committee [21]. Analysis for this work was undertaken as part of Project 43920.

### (b) HCMV serostatus assessment

Baseline serum samples from 9724 UKB participants were selected at random in July 2016 to undergo multiplex serology testing, measuring immunoglobulin G (IgG) antibody responses to specific antigens from 20 infectious agents of interest. Three HCMV antigens (pp28, pp52 and pp150 N terminus) were included within the serology panel, with HCMV seropositivity defined by antibody response median fluorescence intensity (MFI) exceeding a threshold value [22] for at least two out of the three HCMV antigens.

Three HCMV antigens were included in the serology panel (HCMV pp28, pp52 and pp150 N terminus), of which antibody levels to HCMV pp28 were selected as the best measure; pp28 results had a sensitivity of 97% and specificity of 99% versus serostatus according to the gold-standard monoplex HCMV assay [22]. As described in Hamilton *et al.* [20], the seroreactivity threshold for pp28 was set in the gap between the bimodal normal distributions when raw pp28 titres were natural log-transformed. This threshold was set at a natural log-transformed MFI of 6.0. Seropositive patients (ln pp28 ≥ 6.0) were then categorized into low (6.0−7.3), medium (7.4–7.9) and high (≥8.0) tertiles. pp28 antibody levels were also compared between baseline and repeat measurement with regression calculations.

### (c) Measuring cardiovascular outcomes

Inpatient diagnoses were derived from Hospital Episode Statistics for England, Scottish Morbidity Record for Scotland and Patient Episode Database for Wales [23]. CVD was defined as the presence of International Classification of Diseases (ICD) codes corresponding to IHD or stroke. ICD-9 codes (430, 431, 434, 436 for IHD and 410, 411, 412, 413, 414 for stroke) were used to record diagnoses prior to 1996 and ICD-10 codes (I20, I21, I22, I23, I24 and I25 for IHD and I60, I61, I63, I64 for stroke) for diagnoses from 1996 onwards.

### (d) Measuring mortality

CVD- and all-cause mortality were defined using death registry records from the National Health Service Digital for England and Wales, and the Information and the Statistics Division for Scotland. Deaths where the primary cause of death was listed as an external factor, e.g. car accidents or surgical procedures (ICD-10 codes V–Y, *n* = 24), were excluded.

### (e) Covariates

We selected covariates associated with both HCMV serostatus and incident CVD (and mortality) in order to minimize bias caused by confounding factors, including both socioeconomic and health-related measures. Socioeconomic status [24,25] (USA reference) was estimated by the Townsend deprivation score (UK Biobank Field ID: 189), assigned at recruitment to patients based on their home postcode (see [26]). The touchscreen questionnaire on initial assessment provided information on ethnicity [25,27] (Field ID: 21000), level of education [25] (Field ID: 6138), diabetes status [28] (Field ID: 2443 for self-reported diabetes), smoking status [29] (Field ID: 20116) and alcohol consumption [30] (Field ID: 1558), all of which are recognized to predict both risk of HCMV infection, CVD and all-cause mortality. Body mass index (BMI) [31] (Field ID: 21001) and systolic blood pressure (Field ID: 4080) were measured during the physical examination. C-reactive protein (mg l^−1^), low-density lipoprotein (mmol l^−1^) and triglyceride levels (mmol l^−1^) were measured from blood samples using multiple immunoassay and clinical chemistry analysers tested and calibrated to internationally recognized standards. Diabetes status was also assessed through measurement of HbA1c; participants were classified as diabetic if they reported diabetes diagnosed by a doctor, or their measured HbA1c exceeded 48 mmol mol^−1^. Use of aspirin, blood pressure medication or cholesterol medication was hypothesized to be confounders when testing for HCMV serostatus with C-reactive protein (CRP) and CVD or all-cause mortality, replicating analyses completed in the NHANES-III study [7].

Complete case analysis (excluding participants with any missing relevant data) was used for continuous variables, while missing data, 'prefer not to answer' or 'do not know' responses were included as an additional category in categorical variables. Overall proportions of missing data were low: <0.7% for categorical variables and <1.6% for continuous variables.

**Figure 1.**
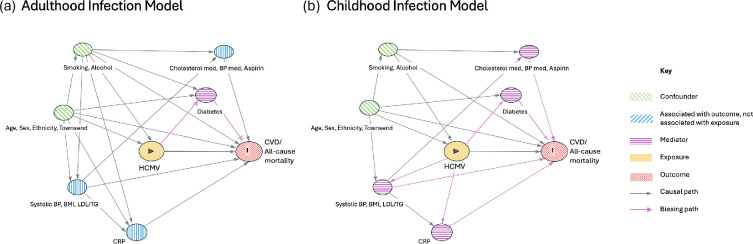
Directed acyclic graphs (DAGs) depicting the relationships between variables in the adulthood (a) and childhood (b) infection models,respectively. Variables with similar interactions are grouped together to improve the readability of the graph. Graphs produced in DAGitty (https://www.dagitty.net/). BMI, body mass index; CVD, cardiovascular disease; BP, blood pressure; LDL, low-density lipoprotein levels; TG, triglyceride levels; CRP, C-reactive protein

We developed two DAGs (see figure 1) linking exposure to HCMV during either childhood or later in adulthood in order to determine in each which covariates were confounders (and hence should be adjusted for) and which were mediators (on the pathway between exposure and outcome) and should not be adjusted for. Assuming infection in childhood, given the hypothesized inflammatory mechanism by which HCMV causes pathology, factors that could lie on the causal inflammatory pathway (hypertension, CRP, low-density lipoprotein (LDL), triglycerides) and those modifying them (blood pressure medication, cholesterol-lowering medication, aspirin) should not be adjusted for. For this model, we considered socioeconomic factors such as Townsend deprivation score and education, alongside smoking and alcohol consumption, to be intrinsically associated with socioeconomic status at birth and during childhood; therefore, they can be assumed to be fixed pre-birth and are valid confounders to adjust for. Assuming infection later in adulthood, we considered some of the possible mediators of HCMV pathology (hypertension, CRP, LDL, triglycerides, aspirin, blood pressure medication and cholesterol medication) to be fixed pre-infection (more likely to be caused by obesity, etc.). According to the adulthood model DAG, no directional path exists between HCMV exposure and CVD via any of hypertension, CRP, LDL or triglycerides; instead, each is independently associated with the outcome (CVD or all-cause mortality). Adjusting for these factors in the adulthood model therefore does not introduce collider bias according to our DAG, but instead reduces variance in the outcome and increasies the precision of results. Similarly, we hypothesized that use of aspirin, blood pressure medication or cholesterol medications again was not causally linked to HCMV exposure, and there was no path on the DAG runs from HCMV exposure to the outcome via use of these medications; therefore, we could adjust for these in our adulthood model to increase precision in results without risking collider bias.

For each individual participant in the UKB, it is not possible to determine when they were infected with HCMV, so the results of applying each model to the whole cohort can demonstrate the effects of adjusting for additional confounders except for length of time since first defined as seropositive against HCMV.

Given that this DAG approach attempted to model exposure risks based on possible age of exposure, we further explored the effect of stratifying the participants by age categories. We also explored the effect of stratifying analyses into groups of smokers and non-smokers.

### (f) Statistical analysis

Comparing HCMV serostatus at baseline and at reassessment for the subset of participants selected to re-attend assessment centres provided data on the validity of assuming that HCMV serostatus remained constant throughout the study. Additionally, variability of HCMV antibody levels was determined by comparing baseline and reassessment data.

Cox proportional-hazards models were used to estimate hazard ratios (HRs) and 95% confidence intervals for developing incident CVD and all-cause mortality by HCMV serostatus. Separate models were performed with IHD, stroke and all-cause mortality as outcomes. Follow-up time was measured in person-years at risk from the date of first recruitment until the date of the first diagnosis of CVD or IHD (or all-cause mortality), date of death, date of last hospital admission (30 September 2021 for England and Wales, 31 October 2021 for Scotland: beyond which data are incomplete), whichever came first. Testing the proportional-hazards assumption using the cox.zph() function in R (https://www.rdocumentation.org/packages/survival/versions/3.8-3/topics/cox.zph) showed no significant violations of the assumption, except for the small number of participants who chose not to answer/did not know whether they took either antihypertensive or cholesterol-lowering medication.

Risk of incident CVD is significantly increased in patients who have previously had IHD or a stroke event [32,33]; therefore, the models measuring CVD events (and IHD and stroke individually) as outcomes were repeated when including and when excluding those participants with prevalent CVD at baseline.

Minimally adjusted models with only age (age at recruitment, in years) and sex as confounders were first tested. Additional sociodemographic and clinical confounders were added to fully adjusted models. Fully adjusted models assuming childhood infection with HCMV controlled for ethnicity (white, non-white), Townsend deprivation score (categorized into tertiles), education level (higher education, secondary school or no education), smoking (never, previous, current), alcohol intake (frequent [weekly or daily], occasional, never drinkers), aspirin use (no, yes), anti-hypertensive medication use (no, yes) and cholesterol-lowering medication use (no, yes). Fully adjusted models assuming adult infection additionally included BMI (kg m^−2^), systolic blood pressure (mmHg), LDL (mmol l^−1^) and triglyceride levels (mmol l^−1^) as confounders. As described in Hamilton *et al.* [20], it is not clear whether CRP acts as a mediator or confounder between HCMV and incident CVD and all-cause mortality; for this analysis, we considered it to be fixed pre-infection for the adult infection model.

Research implicating HCMV infection in the development of type 1 and type 2 diabetes using cohorts at different stages of life [34–37] suggests that diabetes may be a mediator in the relationship between HCMV infection and incident CVD or all-cause mortality; therefore, it was not controlled for in our fully adjusted models.

Smoking is a considerable risk factor for CVD and all-cause mortality; therefore, the hypothesized atherosclerotic effects of HCMV could be associated with an increased hazard ratio (HR) in smokers. We separated participants into smokers (current or previous) and never smokers, applying the survival models to each.

Statistical analysis was performed in R (v. 2023.06.0 + 421) (https://dailies.rstudio.com/version/2023.06.0+421.pro1/) and statistical significance was defined at *p* < 0.05.

## 3. Results

Participants (*n* = 9724) were selected at random to have their samples assayed for antibodies against 20 infectious agents, of which 101 samples were not valid or no longer available for data analysis, and 258 participants only had resurvey data and were therefore excluded. Furthermore, 625 participants had prevalent CVD at baseline. The model assuming infection in childhood adjusted for no continuous variables, resulting in a final sample of 8740 participants. The model assuming infection in adulthood included continuous variables as confounders, for which 377 participants lacked some data and therefore were excluded, leaving 8362 participants with complete data for analysis.

Of the 8740 participants, 57.7% (5040) were HCMV seropositive at baseline. The HCMV seronegative group contained a younger, slightly more male, less ethnically diverse, less deprived and better educated population compared with the seropositive group (see table 1). The seronegative group members were also less likely to smoke or drink alcohol, and had lower systolic blood pressure, aspirin, anti-hypertensive or cholesterol-lowering medication use. There was no significant difference in baseline average LDL, triglyceride or CRP levels.

**Table 1.** Baseline characteristics of the cohort, the HCMV seronegative group and the HCMV-seropositive group. IQR, interquartile range.

characteristics		overall population (*n* = 8740)	HCMV seronegative (*n* = 3700, 42.3%)	HCMV seropositive (*n* = 5040, 57.7%)
age (years; mean, s.d.)		56.5 (8.2)	55.2 (8.3)	57.5 (7.9)
age group (years)	40−49	2200 (25.2%)	1148 (31.0%)	1052 (20.9%)
	50−59	2910 (33.2%)	1231 (33.3%)	1679 (33.3%)
	60−70	3630 (41.5%)	1321 (35.7%)	2309 (45.8%)
sex	male	3716 (42.5%)	1637 (44.2%)	2079 (41.3%)
	female	5024 (57.5%)	2063 (55.8%)	2961 (45.8%)
ethnic background	White	8233 (94.2%)	3634 (98.2%)	4599 (91.3%)
	non-White	466 (5.3%)	53 (1.4%)	413 (8.2%)
	missing	41 (0.5%)	13 (0.4%)	28 (0.6%)
deprivation	least-deprived third	4641 (53.1%)	2051 (55.4%)	2590 (51.4%)
	middle third	2751 (31.5%)	1156 (31.2%)	1595 (31.6%)
	most-deprived third	1340 (15.3%)	490 (13.2%)	850 (16.9%)
	missing	8 (0.1%)	3 (0.1%)	5 (0.1%)
education level	higher education	5224 (59.8%)	2329 (63.3%)	2895 (57.4%)
	secondary school	2033 (23.2%)	898 (24.3%)	1135 (22.5%)
	no education	1387 (15.9%)	446 (12.1%)	941 (18.7%)
	missing	96 (1.1%)	27 (0.7%)	69 (1.4%)
smoking status	never smoker	4926 (56.4%)	2154 (58.2%)	2772 (55.0%)
	ex-smoker	2914 (33.3%)	1170 (31.6%)	1744 (34.6%)
	current smoker	851 (9.7%)	364 (9.8%)	487 (9.7%)
	missing	49 (0.6%)	12 (0.3%)	37 (0.7%)
alcohol intake	never	2018 (23.1%)	804 (21.7%)	1214 (24.1%)
	occasional	6049 (69.2%)	2655 (71.7%)	3394 (67.3%)
	frequent	657 (7.5%)	237 (6.4%)	420 (8.3%)
	missing	16 (0.2%)	4 (0.1%)	12 (0.2%)
medications	aspirin	822 (9.4%)	308 (8.3%)	514 (10.2%)
	blood pressure med	1468 (16.8%)	554 (15.0%)	914 (18.1%)
	cholesterol med	1105 (12.6%)	404 (10.9%)	701 (13.9%)
systolic BP, mmHg, mean (s.d.)		139.8 (19.9)	138.7 (19.6)	140.6 (20.1)
BMI, kg m^−2^, mean (s.d.)		27.2 (4.8)	26.8 (4.7)	27.5 (4.8)
LDL, mmol l^−1^, mean (s.d.)		3.6 (0.9)	3.6 (0.9)	3.6 (0.9)
triglycerides, mmol l^−1^, mean (s.d.)		1.7 (1.0)	1.7 (1.0)	1.7 (1.0)
C-reactive protein, mg l^−1^, median (IQR)		1.3 (0.7−2.7)	1.2 (0.6−2.6)	1.4 (0.7−2.8)

Over a mean follow-up period of 12.7 years, 853 CVD events were reported over the period (1386 if including participants who had prevalent CVD at baseline), of which 190 were fatal events. This included 731 IHD events and 188 stroke events separately. This was an increase of 227 CVD events when compared with the previous analysis of this data [20].

**Figure 2.**
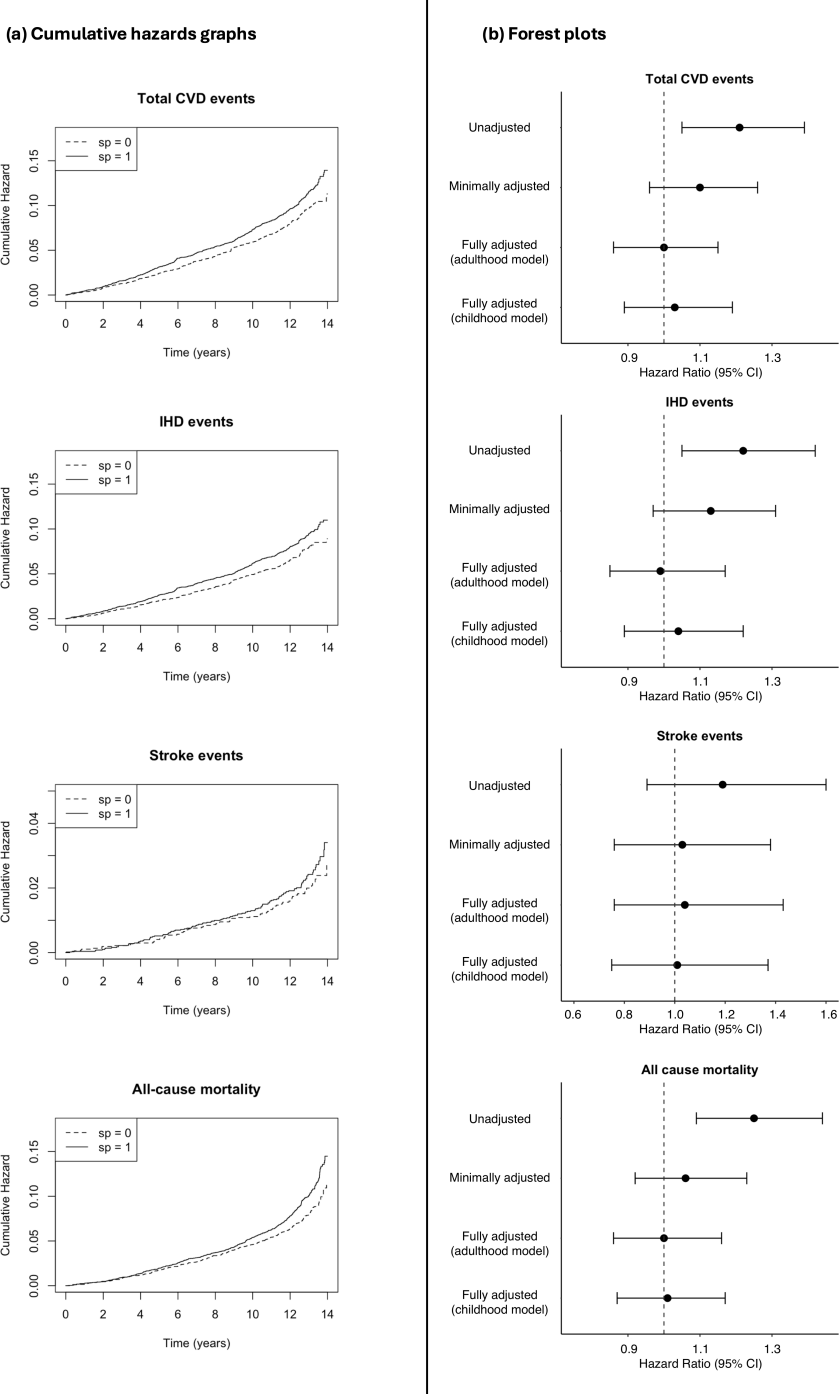
Cumulative hazard graphs (a) and forest plots (b) for all measured outcomes. Red dashed vertical line demonstrates a hazard ratio of 1.0.

Testing for an association between HCMV seropositivity and incident CVD events, an unadjusted Cox proportional-hazards model demonstrated an increased risk (HR 1.21, 95% CI 1.05−1.39, *p* = 0.0068) associated with seropositivity. However, significance was lost when adjusting for age and sex (HR 1.10, 95% CI 0.96−1.26). An exploratory model adjusting for sex alone generated a slightly higher HR (1.24, 95% CI 1.08−1.42) compared with the unadjusted model. When the two separate models were applied and further adjusted for different health-related and sociodemographic confounders, assuming either infection in childhood or adulthood, no association was any longer apparent in either of them (adulthood: HR 1.00, 95% CI 0.86−1.15; childhood HR 1.03, 95% CI 0.89−1.19). Unadjusted models on risk of IHD individually also demonstrated a significant association with HCMV seropositivity (HR 1.22, 95% CI 1.05−1.42), trending to null when adjusting for age and sex (HR 1.13, 95% CI 0.97−1.31) and in the adulthood or childhood infection models, as shown in table 2. Applying the analysis on stroke events individually showed no significance for unadjusted, minimally adjusted, or fully adjusted models. Figure 2 includes Nelson-Aalen graphs displaying difference in cumulative hazard between seropositive and seronegative groups, and Forest plots demonstrating the HRs associated with HCMV seropositivity for each model and each outcome.

**Table 2.** Hazard ratios and 95% confidence intervals calculated for HCMV-seropositive participants compared with seronegative participants for each outcome using unadjusted, minimally adjusted or fully adjusted Cox proportional-hazards models (adulthood (A) or childhood (C) infection model for fully adjusted models).

outcome	model	HR (95% CI)
total CVD events (853)	unadjusted	1.21 (1.05−1.39)
	minimally adjusted	1.10 (0.96−1.26)
	fully adjusted	A: 1.00 (0.86−1.15), C: 1.03 (0.89−1.19)
IHD events (704)	unadjusted	1.22 (1.05−1.42)
	minimally adjusted	1.13 (0.97−1.31)
	fully adjusted	A: 0.99 (0.85−1.17), C: 1.04 (0.89−1.22)
stroke events (188)	unadjusted	1.19 (0.89−1.60)
	minimally adjusted	1.03 (0.76−1.38)
	fully adjusted	A: 1.04 (0.76−1.43), C: 1.01 (0.75−1.37)
all-cause mortality (811)	unadjusted	1.25 (1.09−1.44)
	minimally adjusted:	1.06 (0.92−1.23)
	fully adjusted:	A: 1.00 (0.86−1.16), C: 1.01 (0.87−1.17)

Sensitivity analyses including applying the models without excluding patients who had prevalent CVD at baseline resulted in very similar observations. Fully adjusted HRs in both adulthood and childhood infection models showed no significant association between HCMV seropositivity and total CVD events (adulthood: 0.97, 95% CI 0.87−1.09; childhood: 1.03, 95% CI 0.92−1.15), including when IHD or stroke were the measured outcomes.

Results for all-cause mortality as the measured outcome mirrored those of total CVD, IHD and stroke: unadjusted HR 1.25 (95% CI 1.09−1.44), minimally adjusted HR 1.06 (95% CI 0.92−1.23), fully adjusted HRs 1.00 (95% CI 0.86−1.16) (adulthood), 1.01 (95% CI 0.87−1.17) (childhood).

Increased age (measured as age at recruitment) is associated with both HCMV seropositivity and CVD, as shown in this cohort (mean age of seropositive group: 57.46, mean age of seronegative group: 55.23; mean age of participants who have had a CVD event: 60.00, mean age of participants who have not had a CVD event: 55.72).

Stratifying participants into three categories based on age at recruitment demonstrated significant variations in calculated HRs (applying the fully adjusted models) across the age categories. Among the 40−49-year-old cohort, HCMV seropositivity was significantly associated with total CVD events (adulthood: HR 1.67, 95% CI 1.07−2.62; childhood: HR 1.54, 95% CI 0.99−2.38), while no significant results were shown in either the 50−59 or 60−70-year-old categories (full results in table 3). Estimated HRs calculated for all-cause mortality showed no significant results. It was noted that there were small numbers of outcomes (90 CVD events and 52 all-cause deaths) in the 40−49-year-old age category.

**Table 3.** Hazard ratios and 95% confidence intervals calculated (using adulthood (A) or childhood (C) infection model for fully adjusted models) for risk of total CVD events in seropositive participants versus seronegative participants categorized into groups based on age at recruitment.

age category (years)	HR (95% CI)
40−49	A: 1.67 (1.07−2.62), C: 1.54 (0.99−2.38)
50−59	A: 0.85 (0.65−1.12), C: 0.94 (0.72−1.22)
60−70	A: 1.14 (0.95−1.37), C: 1.16 (0.97−1.38)

Investigating the risk of CVD events or all-cause mortality associated with HCMV seropositivity in smokers (current or previous) versus never smokers demonstrated a raised HR in smokers for both adulthood and childhood infection models in both CVD and all-cause mortality; however, this was not statistically significant in any model (see table 4).

**Table 4.** Hazard ratios and 95% confidence intervals calculated (using adulthood (A) or childhood (C) infection model for fully adjusted models) for risk of total CVD events and all-cause mortality in seropositive participants versus seronegative categorized into groups based on smoking status: smokers (current or previous) or never smokers.

outcome	smoker/non-smoker	HR (95% CI)
total CVD events	non-smokers	A: 0.92 (0.74−1.13), C: 0.98 (0.80−1.20)
	smokers	A: 1.08 (0.88−1.33), C: 1.10 (0.90−1.34)
all-cause mortality	non-smokers	A: 0.90 (0.72−1.14), C: 0.93 (0.74−1.17)
	smokers	A: 1.07 (0.87−1.31), C: 1.06 (0.87−1.28)

HRs associated with total CVD events for low, medium and high HCMV antibody titre tertiles, respectively, were 0.98 (95% CI 0.81−1.19), 1.10 (95% CI 0.91−1.33), 0.99 (95% CI 0.81−1.20) for the adulthood model (*p* for trend according to Schoenfeld residuals = 0.55) and 1.03 (95% CI 0.86−1.25), 1.16 (95% CI 0.96−1.39), 1.00 (0.82−1.21) for the childhood model (*p* for trend = 0.44). No significant relationship was found between HCMV antibody titre tertile and all-cause mortality: HRs generated for low, medium and high tertiles, respectively, were 0.95 (95% CI 0.78−1.16), 1.10 (95% CI 0.90−1.34) and 1.11 (95% CI 0.91−1.34) for the adulthood model and 0.96 (95% CI 0.79−1.17), 1.09 (95% CI 0.90−1.32) and 1.11 (95% CI 0.91−1.34) for the childhood model (see table 5).

**Table 5.** Hazard ratios and 95% confidence intervals calculated (using adulthood (A) or childhood (C) infection model for fully adjusted models) for risk of total CVD events and all-cause mortality in seropositive participants stratified based on natural log of anti-HCMV pp28 IgG antibody titre (low = 6.0–7.3, medium = 7.4–7.9, high ≥ 8.0).

outcome	antibody tertile	HR (95% CI)
total CVD events	low	A: 0.98 (0.81−1.19), C: 1.03 (0.86−1.25)
	medium	A: 1.10 (0.91−1.33), C: 1.16 (0.72−1.22)
	high	A: 0.99 (0.81−1.20), C: 1.00 (0.82−1.21)
all-cause mortality	low	A: 0.95 (0.78−1.16), C: 0.96 (0.79−1.17)
	medium	A: 1.10 (0.90−1.34), C: 1.09 (0.90−1.32)
	high	A: 1.11 (0.91−1.34), C: 1.11 (0.91−1.34)

## 4. Discussion

This study investigated whether HCMV seropositivity (indicating past infection with HCMV) was associated with incident CVD, IHD and stroke individually, or all-cause mortality in a middle- to older-aged UK-based cohort of roughly 8700 participants, specifically accounting for the role of important variables through DAGs. Our results suggest that HCMV seropositivity is not significantly associated with an increased risk of CVD, IHD, stroke or all-cause mortality when adjusting for various relevant sociodemographic and health-related factors. Given the challenge in accounting for the unknown age of acquisition of HCMV, we used an alternative approach to those used previously, adjusting for additional covariates that could be considered confounders assuming HCMV infection in adulthood rather than childhood, including CRP, LDL and triglyceride levels. Overall, these analyses did not demonstrate any further significant deviation from a null effect of HCMV on risk of CVD, IHD, stroke or all-cause mortality. Repeating the same analysis when including participants who had prevalent CVD at baseline also did not show any significance for CVD, IHD or stroke as outcomes. The only analysis that appeared to demonstrate an association between HCMV and CVD was the age-stratified analysis, where the younger 40−49-year-old strata (at recruitment) exhibited a greater risk of CVD events in the HCMV-exposed group; however, the total number of events in this group was low, and there is a risk of this being a false-positive association owing to the number of independent associations tested.

Our analyses of antibody titre tertiles (low, medium or high) did not show any evidence for association with risk of developing CMV, IHD, stroke, or all-cause mortality compared with seronegative individuals when adjusting for relevant confounders. Furthermore, we observed no consistent trend across the tertiles; however, the number of events per tertile was low, widening the 95% CI.

The lack of a significant association between HCMV seropositivity and CVD events in our study mirrors the results of several studies discussed in Wang *et al.* [8], a meta-analysis of 10 prospective cohort studies totalling 34 564 participants and 4789 CVD patients. The complete meta-analysis calculated an overall relative risk of 1.22 (95% CI 1.07−1.38) for CVD events in HCMV-seropositive versus seronegative individuals; however, the meta-analysis acknowledged the inclusion of studies with possible selection bias and limited control for confounding variables. Four of the studies only measured cardiovascular death as their outcome [7,38–40], of which two [38,40] reported significant increases in risk of CVD for seropositive participants only adjusted for sex and/or age. Three of the included studies [41–43] were performed in participants known to either have CVD at baseline or be at considerable risk of developing it.

Our results did not show any significant relationship between HCMV seropositivity and specifically IHD or stroke as outcomes, matching the results shown in a search of the literature. Wang *et al.* [8] reported a pooled relative risk of 1.16 (95% CI 0.95−1.42) for specifically IHD across the three included studies [17,41,42] that measured it as an outcome, although the quality of evidence was reported as ‘very low’ according to GRADE (Grading of Recommendations, Assessment, Development and Evaluation) guidelines. An additional three studies [44–46] reported a non-significant increase in risk of incident IHD associated with HCMV seropositivity, while four others [47–50] calculated a non-significant decrease in risk of incident IHD. Considering stroke as the outcome, Wang *et al.* [8] reported a pooled relative risk of 1.16 (95% CI 0.67−2.01) from the three included studies [41,42,51] that measured it as an outcome, although the quality of evidence was again ‘very low’. A meta-analysis [52] of 12 studies reported no significant association between HCMV IgG seropositivity and risk of incident stroke, calculating summary estimates of 1.40 (95% CI 0.67−2.01) for the six case–control studies and 1.01 (95% CI 0.73−1.39) for the six cohort studies. This evidence was also of very low quality despite the large number of total participants (7424, including 1143 cases of stroke), as 10 out of the 12 included studies had a high risk of bias in one of sufficient adjustment for confounders, selection of participants or reverse causation; in 6 of the 12 studies, HCMV seropositivity was determined post-stroke and 5 out of the 6 case–control studies were retrospective rather than nested in design, giving lesser significance to their overall effect estimate.

Our findings showing no significant association between HCMV seropositivity and all-cause mortality were not consistently replicated throughout the literature. Simanek *et al.* [7] reported an increased risk of all-cause mortality in HCMV-seropositive participants (HR 1.19, 95% CI 1.01−1.41) in a large (14 153 participants) nationally representative population of adults ≥25 years of age within the USA, even when adjusting for age, gender, race/ethnicity, country of origin, education level, BMI, smoking status and diabetes status. Further adjusting for CRP level did not attenuate the risk associated with HCMV seropositivity. This study, however, did not mention excluding participants with prevalent CVD and did not adjust specifically for socioeconomic status (e.g. Townsend score, as we used for the UK). Its population was considerably younger (mean age 46.5 years), with a seronegative group that was significantly younger, more male, White, originating in the USA, higher educated, with a lower BMI and less diabetic compared with its seropositive group, additionally reporting a significantly lower CRP level in the seronegative group. The significant relationship (although with a small number of events) shown between HCMV seropositivity and total CVD events in the 40−49-year-old strata within our study, however, does correlate with the results of Simanek *et al.* [7] (which has a similar average age of cohort): this suggests that HCMV exposure may be associated with CVD outcomes in younger individuals, although the effect is lost in older age. Another large cohort study of participants aged 40−79 years in the UK reported an association between HCMV seropositivity and all-cause mortality (HR 1.12, 95% CI 1.02−1.23) when adjusting for relevant confounders [17]. Two studies among older White populations in Northern Europe, however, did not show a significantly increased risk of all-cause mortality in HCMV-seropositive participants: in 10 122 White community-dwelling adults in the Netherlands and Denmark, they estimated HR 1.00, 95% CI 0.82−1.21 when adjusting for relevant confounders [18], and in 549 participants in Belgium: unadjusted HR 1.17, 95% CI 0.77−1.71 [19].

We did not find any significant dose–response relationships between HCMV antibody titre and either CVD or all-cause mortality as outcomes. Some evidence from the literature suggests that high antibody titre levels are associated with increased risk of CVD events and all-cause mortality. Gkrania-Klotsas *et al.* [53] calculated an increased risk of IHD events among the highest IgG antibody tertile of HCMV-seropositive participants compared with seronegative (HR 1.21, 95% CI 1.04−1.41), even when adjusting for relevant confounders. Several studies reported an increased risk of all-cause mortality in HCMV-seropositive participants with the highest IgG tertile [17,19] compared with seronegative, and Lee *et al.* [54] showed a significant association between HCMV antibody levels (using a survival model with HCMV antibody as a continuous variable) and all-cause mortality (HR 1.04, 95% CI 1.00−1.07).

Our study has a large sample size, with an additional 2−3 years of follow-up compared with Hamilton *et al.* [20] including an increase of 227 total CVD events, 165 IHD events and 44 strokes (discrepancy in addition owing to exclusion of all participants with prevalent CVD at baseline in stroke analysis as opposed to solely excluding those with previous strokes in the former analysis). The UKB has extensive and accurate data enabling us to control for confounding variables in our models without losing any significant number of participants owing to missing data. Measurement of HCMV serostatus via the multiplex serology panel also has high accuracy (specificity of 96.9% and sensitivity of 98.7% compared with the gold-standard reference panels) [22]. The accuracy of hospital inpatient records for measuring CVD events in the UK has also been validated in Kivimäki *et al.* [55].

Our use of different models adjusting for (or not adjusting for) various confounders that have a controversial role in any possible association between HCMV seropositivity and incident CVD or all-cause mortality, including CRP, is a considerable strength of our study. The lack of significant association between HCMV seropositivity and either CVD (including any of its subtypes) or all-cause mortality for the main analyses, even when excluding or including participants with prevalent CVD at baseline, lends further weight to the suggestion that HCMV-seropositive individuals are at no higher risk of any of the measured outcomes than seronegative individuals, when adjusting for relevant confounders.

Although our cohort is large and of comparable size to the largest cohorts in the literature, it has several limitations. First, the UKB population is significantly affected by volunteer bias, consisting of an older, more female, less ethnically diverse cohort living in less socioeconomically deprived areas than the eligible population [56]. Participants were less likely to be obese or smoke, with a lower prevalence of self-reported health conditions including CVD, hypertension, diabetes and chronic kidney disease compared with the general population [56]. HCMV seroprevalence is associated with non-White ethnicity [57,58] and lower socioeconomic status (in studies in the USA [25] and from measurents of HCMV IgG in the Netherlands [59]), therefore, may be lower in the UKB population than in the general population. The lower prevalence of CVD and HCMV in our cohort compared with the general population could introduce collider bias in our analysis and risk generating a null in-sample association, especially if the association between HCMV and CVD events is weak: e.g. if HCMV and CVD were associated with a Pearson’s correlation coefficient of 0.2 in the general population, even selecting just 5% of participants with a lower risk of both characteristics would result in no significant association between them within the study [60]. Our cohort of under 9000 participants may still be too small for our study to have sufficient power to detect any small effect size of HCMV seropositivity on CVD or all-cause mortality.

One of the challenging limitations with both our work and the work of others in this field is the inability to determine when participants became infected with HCMV. Acute HCMV infection is usually asymptomatic or indistinguishable from other mild, acute viral infections. This means that there will always be an unknown duration of prior infection status for individuals testing positive for HCMV exposure at the cross-sectional timepoint and also an unknown state of seroconversion in those individuals defined as seropositive at the cross-sectional timepoint. However, we would expect that the combination of a large sample size, alongside variable age of participants tested at the cross-sectional timepoint and our analyses specifically adjusting for age through stratification should attempt to account for this issue. This unknown timing of infection does mean that we are unable to apply our adulthood and childhood infection models with relevant confounders, specifically depending on their time of infection. The possible inflammatory mechanism by which HCMV could influence the development of CVD or all-cause mortality would imply that the length of carriage of the virus would predict risk of the measured outcomes; however, as we cannot determine this from the data, we are unable to adjust for it as a confounder in our analyses. Data from other cohorts with a wider spectrum of age of recruitment and more longitudinal sampling would help address this issue.

Our analysis of all-cause mortality as the outcome excludes deaths owing to external causes such as accidents; however, possible HCMV-related mortality is likely under-represented as participants may die of other causes before any HCMV-related pathology.

In conclusion, our study continues to demonstrate that, even after careful consideration of covariates, there is little evidence that HCMV exposure increases the risks of CVD or all-cause mortality. However, as existing datasets expand in size and new studies with an increased emphasis on minimizing recruitment bias are acquired, we should continue to ask this question because the current datasets may simply be underpowered to detect an effect, especially if effects are limited to specific subsets of participants such as those acquiring CMV at a younger age, as suggested in our 40−49-year-old age strata (albeit with only 90 CVD events) and Simanek *et al.* [7] (with an average age of 46.5 years). The Multiplex Serology technology used to measure HCMV in UKB is now being applied to an additional 50 000 UKB participants, and the hope is to apply it to all 500 000 participants, as well as other prospective cohorts in China [61], Mexico [62] and Africa [63]. Altogether, if confirmed, such studies could inform the deployment of HCMV vaccines that are currently targeting prevention of congenital infection through maternal vaccination, which may instead be targeted at younger ages to be of benefit to reducing unexpected diseases such as CVD. Future research should also investigate the association of HCMV with other significant disease processes such as cancer [9] and vaccine responses [64] using large databases like the UKB, especially as larger amounts of serology data become available.

## Data Availability

All summary statistics are provided in tables in the manuscript. Raw data are available to all qualified researchers through application to UK Biobank https://www.ukbiobank.ac.uk/. Code is available as supplementary material. Supplementary material is available online [65].
